# The Role of Microvascular Obstruction and Intra-Myocardial Hemorrhage in Reperfusion Cardiac Injury. Analysis of Clinical Data

**DOI:** 10.31083/j.rcm2503105

**Published:** 2024-03-14

**Authors:** Vyacheslav V. Ryabov, Evgenii V. Vyshlov, Leonid N. Maslov, Natalia V. Naryzhnaya, Alexandr V. Mukhomedzyanov, Alla A. Boshchenko, Ivan A. Derkachev, Boris K. Kurbatov, Andrey V. Krylatov, Aleksandra E. Gombozhapova, Stanislav V. Dil, Julia O. Samoylova, Feng Fu, Jian-Ming Pei, Galina Z. Sufianova, Emiliano R. Diez

**Affiliations:** ^1^Department of Emergency Cardiology and Laboratory of Experimental Cardiology, Cardiology Research Institute, branch of the Federal State Budgetary Scientific Institution “Tomsk National Research Medical Center of the Russian Academy of Sciences”, 634012 Tomsk, Russia; ^2^Department of Physiology and Pathophysiology, National Key Discipline of Cell Biology, School of Basic Medicine, Fourth Military Medical University, 710032 Xi'an, Shaanxi, China; ^3^Department of Pharmacology, Tyumen State Medical University, 625023 Tyumen, Russia; ^4^Instituto de Fisiología, FCM–UNCuyo IMBECU - CONICET-UNCuyo, 5500 Mendoza, Argentina

**Keywords:** heart, ischemia, reperfusion, microvascular obstruction, no-reflow

## Abstract

Microvascular obstruction (MVO) of coronary arteries promotes an increase in mortality and major adverse cardiac events in patients with acute myocardial infarction (AMI) and percutaneous coronary intervention (PCI). Intramyocardial hemorrhage (IMH) is observed in 41–50% of patients with ST-segment elevation myocardial infarction and PCI. The occurrence of IMH is accompanied by inflammation. There is evidence that microthrombi are not involved in the development of MVO. The appearance of MVO is associated with infarct size, the duration of ischemia of the heart, and myocardial edema. However, there is no conclusive evidence that myocardial edema plays an important role in the development of MVO. There is evidence that platelets, inflammation, ${\mathrm{Ca}}^{2+}$ overload, neuropeptide Y, and endothelin-1 could be involved in the pathogenesis of MVO. The role of endothelial cell damage in MVO formation remains unclear in patients with AMI and PCI. It is unclear whether nitric oxide production is reduced in patients with MVO. Only indirect evidence on the involvement of inflammation in the development of MVO has been obtained. The role of reactive oxygen species (ROS) in the pathogenesis of MVO is not studied. The role of necroptosis and pyroptosis in the pathogenesis of MVO in patients with AMI and PCI is also not studied. The significance of the balance of thromboxane A2, vasopressin, angiotensin II, and prostacyclin in the formation of MVO is currently unknown. Conclusive evidence regarding the role of coronary artery spasm in the development of MVhasn’t been established. Correlation analysis of the neuropeptide Y, endothelin-1 levels and the MVO size in patients with AMI and PCI has not previously been performed. It is unclear whether epinephrine aggravates reperfusion necrosis of cardiomyocytes. Dual antiplatelet therapy improves the efficacy of PCI in prevention of MVO. It is unknown whether epinephrine or L-type ${\mathrm{Ca}}^{2+}$ channel blockers result in the long-term improvement of coronary blood flow in patients with MVO.

## 1. Introduction

The term “no-reflow” was first proposed by Majno *et al*. (1967) [1]. These investigators found that after ischemia of the rabbit brain lasting 15 min, complete restoration of brain blood flow does not occur. A few years later, Kloner *et al*. [2] could demonstrate that injury of cardiac microvascular vessels is involved in the pathogenesis of no-reflow of the canine heart. In 1985, the no-reflow phenomenon was found in patients with ST-segment elevation myocardial infarction (STEMI) [3]. Investigators reported that thrombolysis could not completely restore coronary blood flow (CBF) in these patients. The duration of chest pain was less than 3 h before admission [3]. Currently, researchers often use the term “microvascular obstruction” (MVO) or the term “the slow flow phenomenon” because complete no-reflow (thrombolysis in myocardial infarction (TIMI) = 0) in the infarct-related coronary artery was detected angiographically in only 5% of patients with acute myocardial infarction (AMI) and percutaneous coronary intervention (PCI), in other patients the incomplete restoration of CBF was observed [4], where TIMI is Thrombolysis In Myocardial Infarction. It should be noted that some investigators suggested that no-reflow could be distinguished as TIMI = 0–1 or TIMI = 0–2 [5, 6, 7, 8, 9]. In this case, the terms “no-reflow” or “MVO” are used as synonyms. There is a correlation between TIMI evaluated angiographically and the MVO size measured by magnetic resonance imaging (MRI) [10, 11]. In a study of patients with AMI and PCI it was found that the duration of cardiac ischemia and infarct size are major determinant of severe MVO [12, 13, 14, 15]. MVO was found in 59% of patients with STEMI + PCI and a 3-h duration of ischemia [13]. If the duration of ischemia was 4–6 h, MVO was found in 72% of patients with STEMI + PCI [13]. The MVO area was measured by MRI [12, 14]. Infarct size in patients with STEMI and MVO was 2-fold larger than in patients without MVO [14]. MVO peaked at 07:00 a.m. [14]. Consequently, infarct size and the duration of ischemia are predictors of the development of MVO. It was reported that the MVO area is 1.9–5.4% of left ventricular (LV) mass in patients with STEMI and PCI according to MRI [11, 16, 17, 18, 19] or 22% of the infarcted myocardium [20]. According to Zia *et al*. [21] the MVO area is 3.1% of the myocardium by MRI. Infarct size was 13–32% of LV mass in patients with STEMI and MVO after PCI by MRI [11, 20, 22]. The intra-myocardial hemorrhage (IMH) area was 3.8% of LV mass in patients with STEMI and MVO after PCI [11]. Currently, the assessment of MVO often uses both angiography and MRI (Table 1, Ref. [5, 7, 9, 10, 11, 12, 13, 14, 16, 17, 18, 21, 22, 23, 24, 25, 26, 27, 28, 29, 30, 31, 32, 33, 34, 35, 36, 37, 38, 39, 40, 41, 42, 43, 44, 45, 46, 47, 48, 49, 50, 51, 52, 53, 54, 55, 56, 57, 58, 59, 60, 61, 62, 63, 64, 65, 66, 67, 68, 69, 70]). However, in recent years, investigators increasingly favor MRI as a more accurate method of assessing MVO. Angiographic parameters are more variable (Table 2, Ref. [5, 7, 9, 10, 11, 18, 23, 24, 25, 26, 27, 28, 29, 30, 31, 32, 34, 35, 36, 38, 39, 43, 44, 71]). Sardu *et al*. [72] found that prediabetes promotes the disorder of acetylcholine-induced coronary vasodilation and major adverse cardiac events in patients with non-obstructive coronary stenosis. Treatment with metformin, an AMP-activated kinase activator, partially restored acetylcholine-induced coronary vasodilation and reduced the incidence of major adverse cardiac events [72]. These data indirectly demonstrated that diabetes can promote the development of MVO and metformin partially reversed this negative effect of diabetes,

Thus, the MVO area is correlated with infarct size and depends on the duration of ischemia.

**Table 1.** **The incidence of MVO and IMH in patients with AMI and STEMI according to angiography, echocardiography and MRI**.

The incidence of MVO and IMH	Reference
The incidence of MVO in patients with STEMI and thrombolysis according to angiographic data is 12%. In the case of AMI + thrombolysis, MVO was found in 19% of patients	[43, 44]
The incidence of MVO in patients with STEMI and thrombolysis according to MRI is 25%	[45]
The incidence of MVO in patients with STEMI and PCI according to angiographic data is 12–74%. In the case of AMI + PCI, MVO was found in 2–70% of patients	[5, 7, 9, 10, 11, 14, 18, 23, 24, 25, 26, 27, 28, 29, 30, 31, 32, 33, 34, 35, 36, 37, 38, 39, 40, 41, 42]
The incidence of MVO in patients with STEMI and PCI according to echocardiographic findings is 50%	[69]
The incidence of MVO in patients with STEMI and PCI according to MRI data is 37–76%. In the case of AMI + PCI, MVO was found in 25% of patients	[12, 13, 16, 17, 21, 22, 46, 47, 48, 49, 50, 51, 52, 53, 54, 55, 56, 57, 58, 59, 60, 61, 62, 63, 64, 65, 66, 67, 68, 70]
The incidence of a combination of MVO + IMH in patients with STEMI and PCI according to MRI findings is 36%–51%	[13, 56, 66]
IMH without MVO was observed in 15% of patients with STEMI and PCI according to MRI	[66]

Note. AMI, acute myocardial infarction included STEMI and Non-STEMI; IMH, intra-myocardial hemorrhage; MRI, magnetic resonance imaging; MVO, microvascular obstruction; PCI, percutaneous coronary intervention; STEMI, ST-segment elevation myocardial infarction. MVO data after thrombolysis or PCI was included.

**Table 2.** **TIMI as index of MVO**.

Index of MVO	Reference
TIMI is 0	-
TIMI is 0–1	[28, 29, 30, 31, 43]
TIMI is 0–2	[5, 7, 9, 10, 11, 18, 23, 24, 25, 26, 27, 32, 34, 35, 36, 38, 39, 44, 71]

Note: MVO, microvascular obstruction; TIMI, Thrombolysis In Myocardial Infarction.

## 2. The Incidence of Microvascular Obstruction, the Mortality Rate, Prognosis

Microvascular obstruction was detected by MRI in 25% of patients with STEMI [45]. Ndrepepa *et al*. [5] reported that MVO was angiographically observed in 29% of patients with STEMI and PCI. According to Mayr *et al*. [65], MVO was found in 56% of patients with STEMI and PCI by MRI. MVO was diagnosed by echocardiography in 50% of patients with STEMI and PCI [69]. It was reported that no-reflow (TIMI = 0–2) was documented in 25% of patients with STEMI and PCI [7]. Microvascular obstruction was found in 25% of patients by angiography (TIMI = 0 or 2) with STEMI and PCI [9]. According to our data, the incidence of MVO is 37% in patients with STEMI and PCI by MRI data [66].

Thus, the incidence of MVO is observed in 25%–56% of patients with STEMI and PCI.

According to Abbo *et al*. [43], the incidence of no-reflow was 66 of 566 (11.6%) patients with AMI and thrombolytic therapy or PCI. They reported that patients with AMI and no-reflow experienced a 10-fold higher incidence of in-hospital death compared to AMI without no-reflow [43]. Cardiovascular events 6 months after AMI in patients with MVO are observed more often than in patients without MVO [45]. The in-hospital mortality rate was 14% in patients with AMI and MVO and only 3% in patients with AMI and without MVO [73]. The no-reflow phenomenon was accompanied by increased mortality for 3 years after AMI [44]. The no-reflow phenomenon was detected angiographically [44]. Patients with STEMI and no-reflow had an increased incidence of in-hospital mortality than patients without no-reflow (TIMI = 0–1) [9]. The no-reflow phenomenon was evaluated angiographically [9]. The mortality rate in patients with STEMI and MVO was greater compared to patients without MVO [14]. Adverse cardiovascular events in patients with AMI and MVO for 2 years after AMI developed more often than in patients with AMI without MVO [45]. MVO is an independent predictor of adverse LV remodeling in patients with STEMI [74]. MVO was evaluated angiographically [74]. In patients with STEMI with PCI, no-reflow (TIMI = 0–1) is a strong independent predictor of the mortality rate for 5 years after AMI [5]. No-reflow was detected angiographically [5]. Microvascular obstruction was usually accompanied by increased myocardial infarct size, a decreased LV ejection fraction, and a high mortality rate for 5 years after AMI [67]. The MVO area was measured by MRI [67]. Microvascular obstruction was associated with adverse cardiac remodeling for 8 months after AMI [68]. The MVO area were measured by MRI [68]. Major adverse cardiac events (MACE) for 6 months after STEMI was documented more often in patients with AMI and MVO [75]. No-reflow was detected angiographically [4]. MVO is a predictor of MACE in patients with STEMI and PCI [22, 59, 60, 61, 64, 76].

In summary, MVO is a common manifestation of AMI, especially in patients with STEMI. Microvascular obstruction is accompanied by a high mortality rate and is associated with MACE.

## 3. The Pathogenesis of Microvascular Obstruction, Analysis of Clinical Data

The highly effective therapy and prevention of MVO are impossible without knowledge of the pathogenesis of this pathology.

### 3.1 Microembolization and Microthrombi

**Fig. 1.**
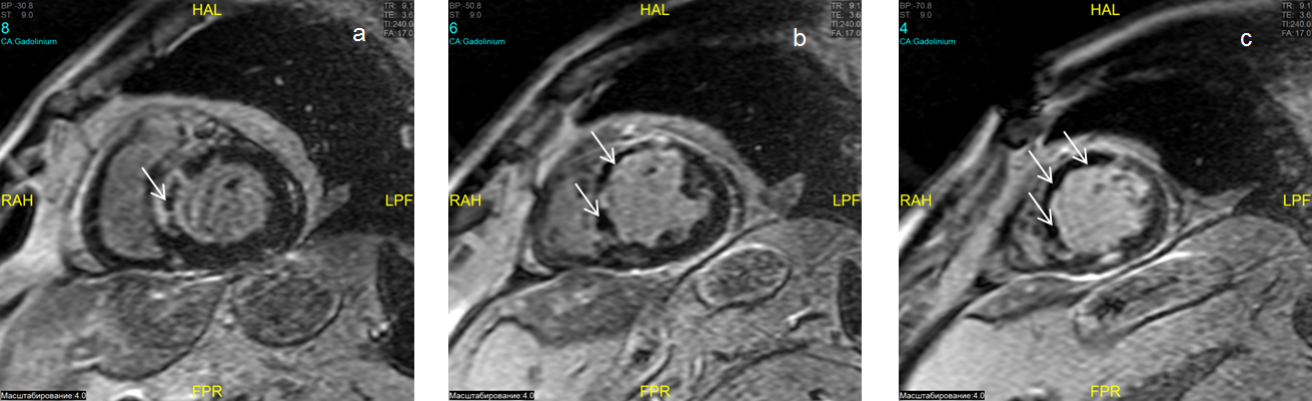
**Microvascular obstruction**. Gadolinium contrast-enhanced cardiac magnetic resonance (DE-CMR) imaging of the basal, middle and apical (a, b, c) short-axis slices. Hypointense areas correspond to no-reflow in the projection of the anterior-septum wall of the left ventricle (LV) of the heart (a, b, c) of the LV in the mode of delayed contrast (inversion recovery sequence). HAL, head anterior left; CA, contrast agent; RAH, right anterior head; FPR, foot posterior right; LPF, left posterior foot; BP, body position; ST, slice thickness.

In a study performed in 2012, MVO was assessed by myocardial blush grade (MBG) in patients with STEMI and PCI [77]. No-reflow was detected angiographically (Fig. 1). Blood samples were drawn from coronary arteries and the aorta for the detection of microparticles. It was shown that the microparticles’ level in the coronary artery is accompanied by MVO. It was concluded that-microparticles could be involved in the development of MVO [77]. This evidence is questionable because these data were not confirmed before by other investigators over the last 10 years. Moreover, it was obtained data that microthrombi is not involved in MVO [10, 11, 18]. Placebo-controlled studies in patients with STEMI + PCI have been performed [10, 11, 18]. The control group received a placebo, whilst alteplase was injected into coronary arteries of patients of the treatment group [10, 11, 18]. The MVO area was measured by MRI [18]. It was found that alteplase did not alter the MVO size measured by MRI [10, 11, 18]. Alteplase had no effect on infarct size but promoted the development of intramyocardial hemorrhage in patients with TIMI flow grade <2 [11]. We found that tenecteplase had no effect on the incidence of MVO.

Microembolization and microthrombi do not play a significant role in the pathogenesis of MVO.

### 3.2 Platelet Aggregation

It has been shown that the microvascular obstruction score is lower in patients with STEMI and PCI who received aspirin than in patients without aspirin [46]. The MVO area was measured by MRI [45]. Patients with STEMI received heparin in the pre-hospital stage following PCI [57]. No-reflow was detected angiographically. Pretreatment with heparin contributed to a decrease in the incidence of no-reflow (TIMI = 0–1) by 13% (*p* < 0.001) [57].

**Fig. 2.**
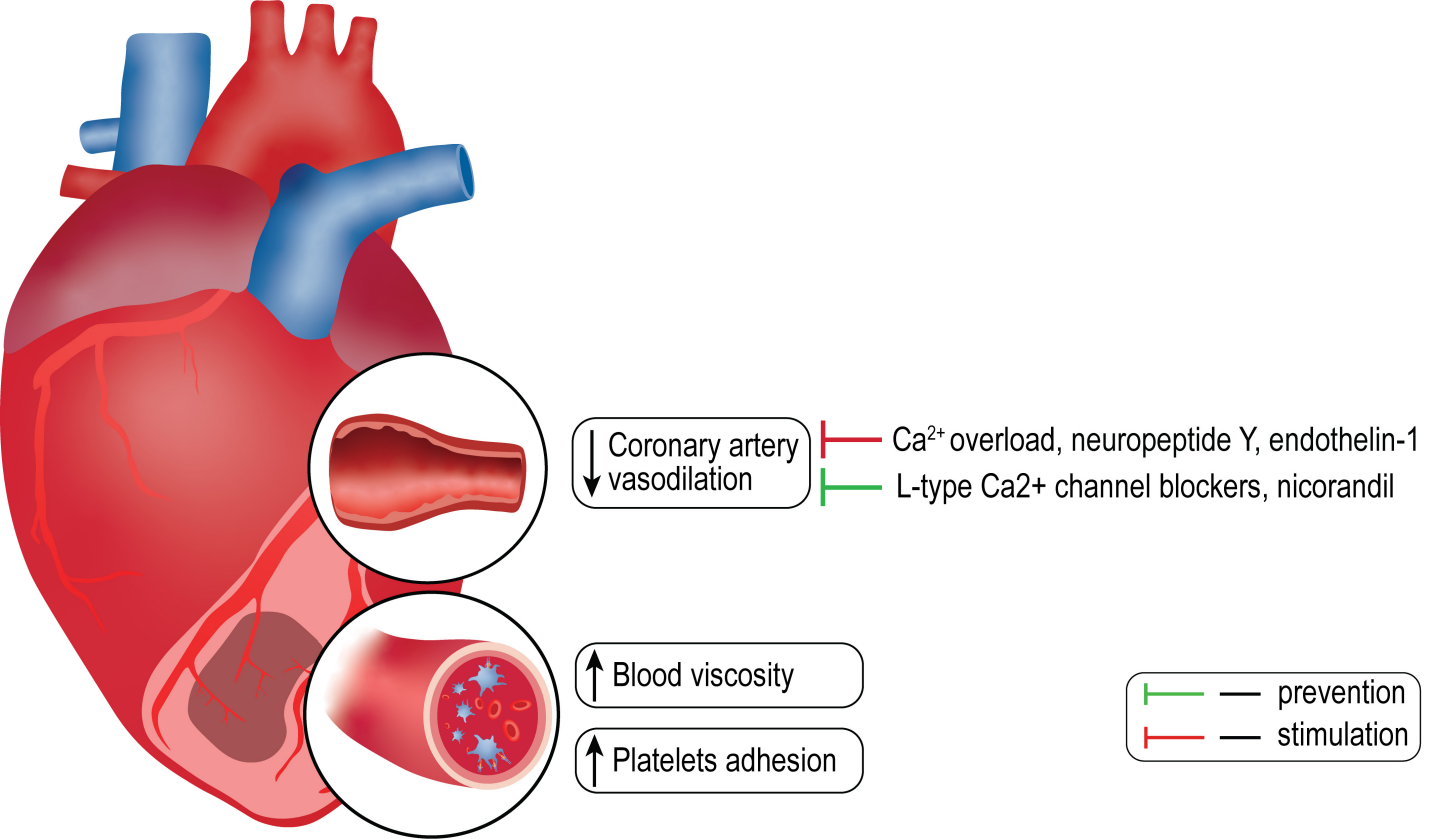
**Some hypothetical pathogenic factors of microvascular obstruction**.

A correlation between the incidence of MVO and ADP-induced platelet aggregation in patients with STEMI and PCI was demonstrated [47]. The MVO area was measured by MRI [47]. A correlation between the incidence of MVO and platelet-neutrophil aggregation was also shown. The incidence of MVO and platelet-monocyte aggregation is also correlated [47]. Consequently, platelets could be involved in MVO (Fig. 2) by releasing a strong vasoconstrictor - thromboxane A2 [78]. Chronic administration of aspirin, a non-selective inhibitor of thromboxane A2 synthesis, resulted in a decrease in the serum concentration of thromboxane B2, a stable metabolite of thromboxane A2 [78]. These findings demonstrate that thromboxane A2 could be involved in MVO. Microvascular obstruction was more frequently observed in patients with STEMI + PCI and high platelet reactivity than in patients with low platelet reactivity [79]. No-reflow was detected as ST-segment regression <50% observed 90 min after PCI [79]. Consequently, platelets could be involved in the development of MVO. The role of thromboxane A2 in the pathogenesis of MVO is required for a study.

### 3.3 Coronary Artery Vasodilation

The role of disturbances of endothelial-dependent vasodilation in MVO remains unclear because the standard endothelial-dependent vasodilator, acetylcholine, was not used in the therapy of MVO in patients with AMI. However, endothelium-independent vasodilators (L-type ${\mathrm{Ca}}^{2+}$ channel blockers, NO donors, nicorandil) were used in the treatment of MVO in minipigs and dogs with coronary artery occlusion and reperfusion [80, 81]. There are data on the use of both endothelium-independent and -dependent relaxation of coronary arteries (β-adrenergic receptor agonists, adenosine) in animals [82, 83, 84]. It was reported that intracoronary administration of verapamil alleviated no-reflow in patients with STEMI [24, 39].

### 3.4 An Increase in Blood Viscosity 

It was shown that acute coronary syndrome is associated with a rise in whole blood viscosity [85]. It was found that whole blood viscosity was higher in patients with STEMI + MVO than in patients with STEMI without MVO [37]. It could be suggested that whole blood viscosity could be involved in the development of MVO in patients with AMI.

### 3.5 Microvascular Obstruction and Adverse Post-Infarction Remodeling of the Heart

It was reported that LV volume was increased in patients with AMI and MVO for six months after AMI, but not in patients with AMI without MVO [38]. No-reflow was detected angiographically [38]. It was demonstrated that infarct size and severe microvascular obstruction were positively correlated with adverse myocardial remodeling for six months after AMI [86]. Other investigators obtained similar evidence of the involvement of MVO in the pathogenesis of adverse post-infarction remodeling of the heart [63, 68, 74, 87]. Adverse left ventricular remodelling occurred in 27% of patients 1 year after STEMI and PCI [76]. Infarct size and MVO were predictors of adverse remodelling according to MRI findings [76]. LV remodelling was defined as ≥10% increase in LV end-diastolic volume from baseline to 4 months after STEMI and PCI by cardiac magnetic resonance (CMR) [88]. LV remodelling was more common in patients with MVO [88]. Aspiration thrombectomy before PCI reduced infarct size, the MVO area and LV remodeling in STEMI patients with a high thrombus burden according to CRM [20].

However, Dregoesc *et al*. [89] could not find a relationship between post-infarction remodeling of the heart and MVO.

Thus, MVO promotes the development of adverse post-infarction remodeling of the heart.

### 3.6 The Role of Inflammation in Microvascular Obstruction 

It was reported that inflammation is involved in ischemia reperfusion (I/R) cardiac injury [90, 91].

It was shown that a high MVO score positively correlated with a rise in the plasma concentration of C-reactive protein (CRP), plasma leukocytes, and peak value of creatine kinase and negatively correlated with high LV ejection fraction in patients with STEMI and PCI [46, 65]. The peak of ${\mathrm{CD14}}^{+}$CD16- monocytes, total monocytes, and total neutrophils in STEMI was higher in patients with MVO than in patients without MVO [48]. The high CRP level at admission could be a predictor of MVO in patients with STEMI [12, 23, 70]. The high plasma concentration of interleukin-6 is also a predictor of MVO in patients with STEMI and PCI [49]. The serum interleukin-18 level was higher in patients with STEMI and MVO than in patients without MVO [92]. We found that the plasma CRP concentration in patients with STEMI + PCI and MVO was 13-fold higher than in patients without MVO on the seventh day after admission.

Consequently, C-reactive protein and interleukins could be involved in the pathogenesis of microvascular obstruction.

### 3.7 The Role of Ca2+ Overload and Reactive Oxygen Species in the Formation of Microvascular Obstruction 

The role of reactive oxygen species (ROS) in the pathogenesis of no-reflow has not been studied before in patients with AMI. The L-type ${\mathrm{Ca}}^{2+}$ channel blockers resulted in endothelium-independent relaxation of coronary arteries [80, 93]. Intracoronray infusion of verapamil mitigated MVO in patients with STEMI [24, 39]. It could be hypothesized that microvascular spasm and ${\mathrm{Ca}}^{2+}$ overload of vascular smooth muscles are involved in the formation of MVO. Consequently, it is possible that coronary artery spasm participates in the pathogenesis of MVO.

### 3.8 Could Nitric Oxide Mitigate MVO? 

Intracoronary administration of sodium nitroprusside, a NO donor, improved the TIMI flow grade in patients with AMI and PCI [94, 95]. However, other investigators reported that intracoronary administration of sodium nitroprusside did not alter CBF in patients with AMI and PCI [25, 26, 50]. Amit *et al*. [25] evaluated no-reflow by ST-segment elevation resolution. Niccoli *et al*. [26] used TIMI grade. Nazir *et al*. [50] used MRI. These studies included larger groups of patients with AMI and PCI, therefore their results are more significant. Consequently, these data demonstrate that coronary artery spasm is not involved in the pathogenesis of MVO.

### 3.9 The Role of Endothelins and Neuropeptide Y in the Development of MVO 

It has been reported that the concentration of endothelin-1 in coronary sinus plasma was 1.7 pmol/L in patients with stable angina and 3.0 pmol/L in patients with AMI [96]. The plasma concentration of endothelin-I in the aorta was higher in patients with AMI than in patients with angina [96]. Microvascular obstruction was evaluated in patients with STEMI and PCI (n = 128) by MRI [51]. The plasma endothelin-1 level on admission was associated with MVO and the mortality rate [51]. These data demonstrated that a rise in the concentration of endothelin-1 in blood could contribute to MVO development. Neuropeptide Y (NPY) is also a strong vasoconstrictor that is released from sympathetic terminals [97]. It was reported that intracoronary administration of NPY resulted in coronary artery spasm in volunteers [98]. The plasma NPY level was higher in patients with STEMI and no-reflow (TIMI = 0–2) than in patients without MVO [6]. In contrast, Herring *et al*. [52] did not find differences in TIMI flow score between patients with high NPY levels and patients with the low NPY levels in coronary sinus blood in patients with STEMI. However, the microcirculatory resistance was higher in patients with high NPY levels in coronary sinus compared to patients with a low concentration of NPY [52].

These data demonstrate that NPY could be involved in the formation of MVO. However, correlation analysis between NPY and endothelin-1 levels and the no-reflow area has not been performed, therefore further studies on the role of NPY and endothelin-1 in the pathogenesis of MVO are required.

### 3.10 The Role of Vasopressin in MVO

It was reported that intravenous injection of arginine vasopressin resulted in coronary artery spasm and ST elevation in rats [99]. Vasopressin triggered a contractile response of isolated coronary arterioles isolated from the heart of patients undergoing cardiac surgery [100]. However, a role for vasopressin in the formation of MVO has not previously been studied.

### 3.11 The Role of Na+/H+ Exchanger in MVO

${\mathrm{Na}}^{+}$/$H^{+}$ exchanger inhibitors were not used before for therapy or prevention of the appearance of MVO in patients with AMI therefore a role of ${\mathrm{Na}}^{+}$/$H^{+}$ exchanger in the development of MVO in patients with AMI is unknown.

### 3.12 The Involvement of β-Adrenergic Receptors (β-AR) in the No-reflow Phenomenon

Intracoronary administration of epinephrine reportedly completely reverses no-reflow in 9 of 12 patients with STEMI and PCI [27]. It should be noted that this group was too small, thereby it is unclear whether β-AR agonists can alleviate MVO. Intracoronary infusion of epinephrine significantly improved CBF in patients with STEMI + PCI and no-reflow (TIMI = 0–1) [28]. The improvement of CBF in patients with STEMI + PCI and no-reflow (TIMI = 0–1) in patients with intracoronary infusion of epinephrine was also shown [29]. MACE after STEMI (1 year) was lower in patients who received epinephrine compared to patients who received adenosine [29]. Mini-pigs were subjected to coronary artery occlusion (CAO) for 3 h and reperfusion for 1 h [40]. Pretreatment with the β_1_- and β_2_-AR antagonist propranolol had no effect on the MVO area in pigs with CAO (3 h) and reperfusion (1 h) [40].

The role of endogenous epinephrine in the prevention of MVO in patients with AMI and PCI remains unclear. Correlation analysis of the MVO size and treatment with the β_1_-antagonists in patients with AMI and PCI is required.

### 3.13 The Involvement of Angiotensin II in Microvascular Obstruction

The angiotensin II receptor antagonists were not used before for the treatment of no-reflow, therefore a role for angiotensin II in MVO remains unclear.

### 3.14 The Role of Adenosine in Microvascular Obstruction

Intracoronary infusion of adenosine decreased the incidence of MVO in patients with AMI and PCI [29, 30, 71]. These investigators detect MVO by angiography [29, 30, 71]. Consequently, adenosine could alleviate MVO.

### 3.15 The Role of Diabetes in Microvascular Obstruction

It has been reported that diabetes contributes to I/R cardiac injury [72]. Hyperglycemia has been reported to be accompanied by MVO in patients with diabetes and AMI [16, 31, 53]. Iwakura *et al*. [31] used intracoronary myocardial contrast echocardiography to detect the no-reflow area. Jensen *et al*. [16] and Ota *et al*. [53] used MRI to measure the MVO area. We also found that a combination of MVO and intramyocardial hemorrhage is more common in patients with hyperglycemia and diabetes mellitus. We used MRI to measure the MVO area.

However, the MVO area can be identical in patients with diabetes and without diabetes [21]. This was shown using MRI [21]. Investigators did not analyze the interaction between type 2 diabetes, insulin-dependent diabetes, the incidence of MVO, and the MVO area. The molecular mechanism of aggravation of MVO by diabetes remains unclear.

### 3.16 The Role of $K_{\text{ATP}}$ Channels in Microvascular Obstruction 

Pinacidil, an ATP-sensitive $K^{+}$ channel ($K_{\text{ATP}}$ channel) opener, triggers endothelium-dependent vasodilation of coronary arteries [81]. Nicorandil, a NO donor and the $K_{\text{ATP}}$ channel opener, resulted in endothelium-independent coronary artery vasodilation [81]. Nicorandil was shown to decrease the incidence of MVO in patients with AMI [101]. However, sodium nitroprusside, a NO donor, was not effective against no-reflow in patients with AMI and PCI [25, 26, 50]. Thus, it could be proposed that nicorandil resulted in coronary artery vasodilation through $K_{\text{ATP}}$ channel opening in these vessels, but not through a rise in the NO level. Unfortunately, the impact of other $K_{\text{ATP}}$ openers on MVO has not been studied before.

### 3.17 The Role of Myocardial Edema in Microvascular Obstruction

There are MRI data to suggest that MVO is accompanied with interstitial edema [10, 11, 18, 21, 50, 52, 54, 55, 56, 58, 102, 103, 104]. This pathology is observed in 50% of patients with STEMI and PCI [50]. Edema could induce extrinsic compression of coronary arteries and trigger MVO. Chen *et al*. [104] found that Myocardial Extracellular Volume Fraction was larger in patients with MVO and IMH by MRI. Myocardial edema in patients with STEMI and MVO was greater than in patients without MVO (*p* < 0.01) by MRI [105]. However, decongestants have not been used to treat MVO in clinical and experimental studies. Therefore, the significance of edema in the pathogenesis of MVO remains unknown.

Thus, microembolization and microthrombi do not play a significant role in the development of MVO. MVO promotes the development of adverse post-infarction remodeling of the heart. Platelets, increased blood viscosity, vasoconstriction, inflammation, NPY, endothelin-1, and myocardial edema could be involved in the pathogenesis of MVO. However, their role in the development of MVO requires further studies because data on their involvement in MVO formation are preliminary and need clarification. The diabetes-induced aggravation of MVO remains unclear.

## 4. Intra-Myocardial Haemorrhage

Microvascular obstruction is often accompanied by intra-myocardial hemorrhage. A combination of MVO and IMH was found in 35–51% of patients with STEMI and PCI, where the hemorrhage area was about 3% of the LV mass [13, 19, 22, 56, 58, 66]. MVO and IMH areas were measured by MRI mass [13, 56, 66]. IMH without MVO was observed in 15% of patients with STEMI and PCI mass [66]. It was reported that the IMH area reached a maximum 24 h after the restoration of coronary perfusion and was about 4% of the left ventricle in pigs with coronary artery occlusion (CAO, 40 min) and reperfusion (24 h), while the maximum MVO area peaked in these pigs was 120 min after the restoration of coronary perfusion [102]. MVO and IMH areas were evaluated by MRI [102]. The largest area of IMH was identified in rats subjected to a 90-minute CAO followed by 48 hours of reperfusion [106]. The IMH area was measured in rats by MRI [106]. The onset of microvascular obstruction precedes the destruction of microvessels and the subsequent emergence of IMH. The presence of IMH was linked to poorer outcomes and the development of unfavorable ventricular remodeling. In patients with AMI, a larger IMH area was associated with longer ischemic durations and delayed reperfusion events [107]. Intra-myocardial hemorrhage was developed in pigs after a 40–120-min CAO and followed reperfusion [102]. According to Ma *et al*. [13] IMH did not develop before successful reperfusion of the heart and IMH size was correlated with infarct size and the MVO area. Investigators used CMR for the measurement of MVO and IMH areas. Intra-myocardial hemorrhage often develops in STEMI patients who have wider and deeper Q waves [108]. Anticoagulant and antiplatelet therapy could contribute to the occurrence of IMH in patients with AMI and PCI [108]. It was reported that the use of alteplase in patients with STEMI and PCI promotes an appearance of IMH [11]. A combination of MVO and IMH was observed in 36%–44% of patients with STEMI and PCI according to MRI data [13, 56, 66]. The pathogenesis of IMH remains unclear. It was reported that patients with IMH had higher CRP, inteleukin-6, fibrinogen, and neutrophils levels compared to patients without IMH [58, 88]. We found that the appearance of IMH is accompanied by an increase in the plasma CRP level was 13-fold in patients with STEMI and MVO. The role of inflammation in the development of IMH requires further investigation.

In summary, IMH is a common manifestation of AMI, especially in patients with STEMI. It is possible that inflammation is involved in the pathogenesis of IMH.

## 5. Reperfusion Therapy for Microvascular Obstruction

In our opinion, microvascular obstruction could be a target for the treatment of reperfusion cardiac injury. In recent years, much attention has been paid to dual antiplatelet therapy (DAPT, three possible combinations: aspirin and clopidogrel; aspirin and prasugrel; aspirin and ticagrelor) for the prevention of MVO [76, 109]. Some investigators performed DAPT in 97% of patients with STEMI and PCI [76].

The incidence of MVO in patients with STEMI and PCI receiving the ${\mathrm{P2Y}}_{12}$ antagonist clopidogrel was 66%, and in patients receiving the ${\mathrm{P2Y}}_{12}$ antagonists prasugrel or ticagrelor the incidence of MVO was 49% [54]. The MVO area was measured by CMR [54]. The glycoprotein IIb/IIIa inhibitor tirofiban was administered intravenously or intracoronally to patients with STEMI and PCI [17]. All patients received clopidogrel and aspirin before PCI. Intracoronary administration of tirofiban reduced the incidence of MVO compared to intravenous injection of tirofiban [17]. Investigators used MRI to measure the MVO area by MRI [17]. The efficacy of DAPT depends on inhibition of platelet aggregation, therefore assessingf the effect of DAPT on platelet aggregation is required. Massalha *et al*. [109] reported that hyporesponsiveness to aspirin or ${\mathrm{P2Y}}_{12}$ receptor inhibitor agents and demonstrated in 29% of patients with STEMI and PCI. Decreased platelet response to DAPT was accompanied by a greater extent of MVO [109].

It has been reported above that intracoronary infusion of adenosine prevents the appearance of MVO in patients with AMI and PCI [30, 32, 71]. Nevertheless, Niccoli *et al*. [26] demonstrated that the intracoronary delivery of adenosine did not influence the occurrence of MVO in a cohort of patients (n = 160) with STEMI who underwent PCI.

Nazir *et al*. [50] also could not find an improvement in MVO in patients (n = 168) with STEMI and PCI. It should be noted that adenosine can aggravate ischemic/reperfusion cardiac injury in patients with AMI through triggering coronary steal [110]. Consequently, adenosine cannot be recommended for the treatment of AMI and MVO.

It was reported above that sodium nitroprusside, a NO donor, did not alter the MVO area [25, 26, 50]. However, nicorandil, a NO donor and $K_{\text{ATP}}$ channel opener, prevents the appearance of MVO by 50% in patients (n = 81) with AMI [101]. Combined intracoronary infusion of adenosine and nicorandil reduced the incidence of no-reflow (TIMI = 0–1) by 40% [33]. No-reflow was evaluated angiographically [33]. Intracoronary infusion of nicorandil reduced the incidence of MVO (TIMI = 0–2) in patients (n = 170) with STEMI and PCI compared to placebo [8].

In dogs with intact coronary arteries and no myocardial hypoxia, β-adrenergic receptor agonists were found to enhance CBF [111]. Nonetheless, when norepinephrine was infused intracoronarily in dogs with coronary stenosis, it led to increased myocardial oxygen consumption and myocardial hypoxia [112]. Administering the β1- and β2-adrenergic receptor agonist isoproterenol (0.1 µg/kg/min) intravenously during coronary artery occlusion and reperfusion in rabbits led to an enlargement of infarct size [113]. This suggests that the use of β-adrenergic receptor agonists in patients with AMI might exacerbate heart I/R injury. It’s worth noting, however, that these findings didn’t rule out the potential of a clinical investigation into the effectiveness of epinephrine intracoronary infusion as a therapy for MVO [28]. It was reported that intracoronary infusion of epinephrine decreases the MVO area in patients with AMI and PCI compared to patients without epinephrine [28]. These data were confirmed by Darwish *et al*. [29]. It should be noted that both groups of investigators did not evaluate the effect of epinephrine on the serum troponin I or creatine kinase levels, thereby it is unclear whether epinephrine can aggravate reperfusion cardiac injury or prevent reperfusion damage. In addition, it should be noted that these studies were not double-blind or placebo controlled.

Consequently, epinephrine could be used for therapy of MVO, but it should be evaluated for its negative effects.

L-type ${\mathrm{Ca}}^{2+}$ channel blockers result in vasodilation of coronary arteries [80, 93]. Intracoronary infusion of verapamil after administration of nitroglycerin improved TIMI flow grade in 89% of patients with AMI and PCI [39]. Intracoronary administration of verapamil decreased the MVO area in patients with AMI and PCI [24, 34, 35]. Intracoronary infusion of the L-type ${\mathrm{Ca}}^{2+}$ channel blocker nicardipine improved TIMI flow grade in 71 of 72 patients with AMI and PCI [41]. Verapamil alleviated no-reflow (TIMI = 0–1) in STEMI + PCI patients [36]. The ability of ${\mathrm{Ca}}^{2+}$ channel blockers to improve TIMI flow grade in patients with STEMI and PCI was confirmed by other investigators who used MRI to evaluate MVO [46]. Co-administration of nicardipine, adenosine, and nitroglycerine reversed no-reflow in patients with AMI and PCI [42].

Consequently, L-type ${\mathrm{Ca}}^{2+}$ channel blockers can be used for the treatment and prevention of MVO. The aforementioned data were summarized in Table 3 (Ref. [8, 17, 24, 25, 26, 28, 29, 30, 32, 33, 34, 35, 36, 39, 41, 50, 54, 71, 101, 109]).

These data demonstrated that clopidogrel, ticagrelor, and nicorandil reduce the incidence of MVO (Table 3). Epinephrine, verapamil, and nicardipine decrease the MVO area (Table 3).

**Table 3.** **The efficacy of reperfusion therapy for microvascular obstruction in patients with AMI**.

Drugs	Effects	Reference
Prasugrel	Incidence of MVO ↓	[54]
Ticagrelor	Incidence of MVO ↓	[54]
Tirofiban	MVO area ↓	[17]
DAPT	MVO area ↓	[109]
Adenosine	Incidence of MVO ↓	[30, 32, 71]
Adenosine	Incidence of MVO no effect	[26, 50]
Nitroprusside	MVO area no effect	[25, 26, 50]
Nicorandil	Incidence of MVO ↓	[8, 33, 101]
Epinephrine	MVO area ↓	[28, 29]
Verapamil	MVO area ↓	[24, 34, 35, 36, 39]
Nicardipine	MVO area ↓	[36, 41]

Note. MVO, microvascular obstruction; AMI, acute myocardial infarction; DAPT, dual antiplatelet therapy.

## 6. Unresolved Issues

Thus, MVO could be the result of an imbalance between vasodilation and vasoconstriction. Microvascular obstruction could be the result of coronary microvascular injury and, above all, the result of endothelial cell damage. It could be an inflammation injury of coronary microvessels. However, anti-inflammatory agents, for example, glucocorticoids have not been used before for treatment of MVO. Therefore, we cannot evaluate a role for inflammation in microvascular injury in patients with AMI and PCI.

Many questions are still waiting for an answer. It is unclear whether there is a role for endothelial cell injury in the pathogenesis of MVO in patients with AMI and reperfusion of the heart. There is no indisputable evidence of the involvement of inflammation in the development of MVO. A role for ROS in the pathogenesis of MVO is also yet to be studied. The role of necroptosis and pyroptosis in the pathogenesis of MVO in patients with AMI and PCI is also not studied. The role of thromboxane A2, vasopressin, angiotensin II, disturbances of nitric oxide production and prostacyclin synthesis in the formation of MVO was not studied before. The role of neuropeptide Y and endothelin-1 in the development of MVO is required in further investigations. It is unclear of the role of coronary artery spasm in the formation of MVO. It was reported that sodium nitroprusside, a donor NO and endothelium-independent vasodilator, did not improve CBF in patients with AMI and PCI. However, verapamil, an L-type ${\mathrm{Ca}}^{2+}$-channel blocker and endothelium-independent vasodilator, mitigates MVO in patients with AMI and PCI. It is possible that smooth muscle cells in coronary arteries lost sensitivity to nitric oxide in patients with MVO. The vasodilator effect of nicorandil, a NO donor and $K_{\text{ATP}}$ channel opener, could be mediated via $K_{\text{ATP}}$ channel opening in smooth muscle cells in coronary arteries.

## 7. Conclusions

Platelets could be involved in the development of microvascular obstruction in patients with AMI and PCI. ${\mathrm{Ca}}^{2+}$ overload of vascular smooth muscle cells in coronary arteries also participates in the pathogenesis of MVO. The duration of ischemia and infarct size are predictors of the appearance of MVO. Intramyocardial hemorrhage was found in 41–50% of patients with STEMI and PCI. Indirect evidence for the involvement of inflammation in the formation of MVO has also been obtained. Increased blood viscosity could contribute to the appearance of MVO. No-reflow and slow flow promote the formation of adverse myocardial remodeling. Probably endogenous catecholamines did not participate in the development of MVO in patients with AMI and PCI. Adenosine and sodium nitroprusside are not able to prevent the appearance of MVO. Intracoronary infusion of nicorandil could be used for the therapy of MVO. Glycoprotein IIb/IIIa inhibitor tirofiban and ${\mathrm{P2Y}}_{12}$ antagonists are low effective in the prevention and treatment of MVO. L-type ${\mathrm{Ca}}^{2+}$ channel blockers could be used for the treatment of MVO. Dual antiplatelet therapy improves the efficacy of PCI in the prevention of MVO.
